# A Diet Containing Animal Source Protein as Fresh, Lean Beef Is More Well Liked and Promotes Healthier Eating Behavior Compared with Plant-Based Alternatives in Women with Overweight

**DOI:** 10.1016/j.cdnut.2024.104415

**Published:** 2024-07-14

**Authors:** Morgan L Braden, Jess A Gwin, Heather J Leidy

**Affiliations:** 1 Department of Nutritional Sciences and Department of Pediatrics, University of Texas at Austin, Austin, TX, United States; 2 Military Nutrition Division, U.S. Army Research Institute of Environmental Medicine, Natick, MA, United States

**Keywords:** plant-based protein, animal-based protein, satiety, food intake, ingestive behavior

## Abstract

**Background:**

Despite limited evidence from intervention trials, replacing animal-source protein-rich foods with plant alternatives continues to be recommended as part of a healthy dietary pattern.

**Objectives:**

The objective of this study was to examine whether a diet containing fresh, lean beef elicits greater satiety, reduces ad libitum food intake, and is more acceptable compared with a diet containing plant alternatives in women with overweight.

**Methods:**

Seventeen women with overweight (mean ± SEM, age: 33 ± 1 y; BMI: 27.8 ± 0.1 kg/m^2^) completed an acute, tightly controlled, crossover design study. Participants were provided with eucaloric, isonitrogenous diets (15% of daily intake as protein) containing either 2 servings/d of fresh lean beef (BEEF) or plant equivalents (PLANT) for 7 d/pattern. During day 6 of each pattern, the participants completed a 10-h controlled-feeding, clinical testing day, which included repeated appetite and satiety questionnaires and blood sampling to assess pre- and postprandial plasma peptide YY (PYY) and GLP-1 across the day. On day 7, the participants completed a free-living testing day in which they consumed their respective protein foods and were provided with additional carbohydrate- and fat-rich foods to consume, ad libitum, during each eating occasion. Energy and macronutrient composition were assessed. A 2- to 3-wk washout period occurred between patterns.

**Results:**

No differences in daily satiety were detected between patterns. During the ad libitum testing day, 24-h food intake was not different between patterns (BEEF: 2714 ± 219 compared with PLANT: 2859 ± 147 kcals/d), BEEF led to fewer carbohydrates consumed compared with PLANT (338 ± 34 compared with 370 ± 22 g/d, *P* < 0.05), especially as sugar (169 ± 73 g compared with 186 ± 57 g, *P* = 0.05). Furthermore, BEEF was more well liked (i.e., higher flavor, texture, and acceptability) compared with PLANT (all, *P* < 0.05).

**Conclusions:**

Although satiety was similar between patterns, the consumption of animal-source protein-rich foods, such as fresh and lean beef, was more well liked and resulted in voluntary reductions in total carbohydrate and sugar intake in middle-aged women with overweight during a single ad libitum testing day.

This study was registered at clinicaltrials.gov as NCT02614729.

## Introduction

Plant-based diets have become popular in the United States [1]. Although not specifically defined by the Dietary Guidelines for Americans (DGAs), some research groups define “plant based” or, more recently “plant forward” to mean a dietary pattern that consists primarily of foods coming from plants [2]. Previous DGAs have supported this dietary pattern through specific recommendations to shift from eating animal-source protein foods, especially red and processed meat, to plant-based alternatives, such as beans, peas, and lentils [3]. However, the majority of Americans continue to include animal-source foods within their respective dietary pattern, potentially as a result of taste, versatility, cultural traditions, and familiarity [4]. Even most individuals who say they habitually follow a plant-based (vegetarian) diet, reported consuming meat, poultry, and fish [5]. This brings into question how feasible it is to replace all animal-source foods with plant-based alternatives and whether there is convincing evidence that this shift is appropriate from a nutrient adequacy, acceptability, or health perspective.

Another related aspect to consider is whether plant- and animal-source protein foods are nutritionally interchangeable. The DGAs use protein-ounce equivalents when recommending protein substitutions [3]. Specifically, 1 ounce of lean meat or poultry is equivalent to ¼ cup cooked beans, 1 tablespoon of peanut butter, 2 ounces of tofu, etc. Yet, a review completed by Gwin et al. [6] assessing protein quality and essential amino acid (EAA) content reports that animal and plant sources, in particular those listed as example DGA equivalents, are typically not matched for energy content, total protein, or EAA content—all of which impact the functional efficacy of protein foods [6].

Furthermore, the state of the evidence is potentially limited by the type of animal- and plant-based foods and beverages assessed. Protein source comparison studies typically include isolated proteins (e.g., whey, soy, pea, casein) [7], [8], [9], [10]. Although most studies illustrate greater skeletal muscle protein synthesis [11], [12], [13], [14] and/or satiety [15], [16], [17], [18], [19], [20] following the acute consumption of isolated animal- compared with plant-based proteins less is known regarding the consumption of whole animal-source protein foods compared with plant-based alternatives within a meal-matrix paradigm (i.e., total meal effects).

Thus, the primary aim of this study was to examine whether a diet containing fresh, lean beef elicits greater overall satiety, as assessed by perceived fullness across the day, and reduces ad libitum food intake compared with a diet containing plant alternatives in healthy women with overweight. Secondary analyses included the assessment of changes in overall hunger, desire to eat, and prospective food consumption. Exploratory analyses included the assessment of palatability and acceptability of the diets. Beef was used for this comparison because it is a widely consumed whole protein food in the United States [21]; in addition, many plant-based alternatives are generally created to resemble beef.

## Methods

### Study participants

The study was approved by the University of Missouri Health Sciences Institutional Review Board, and all procedures were followed in accordance with the ethical standards of the Institutional Review Board. Healthy women with overweight (BMI: 27.8 ± 0.1 kg/m^2^), aged 33 ± 1 y were recruited from January 2014 to May 2015 in Columbia, MO. Having been informed of study procedures and risks, 17 participants provided written consent. The participants received a stipend for completing all study procedures.

### Experimental design

Two full-feeding, tightly controlled crossover studies were originally completed to examine the effects of red meat consumption on appetite control, satiety, and ad libitum intake. Both studies varied in red meat consumption, total protein intake, and calories. Within this article, participants were provided with eucaloric, isonitrogenous diets “standard protein” (15% of daily intake as protein) containing either 2 servings/d of beef (BEEF) or plant equivalents (PLANT) for 7 d/pattern. During the first 5 d of each pattern, the participants were provided with the respective breakfast, lunch, and dinner meals plus an evening snack consumed at home or work. Day 6 of each diet was a tightly controlled 10-h testing day consisting of questionnaires and blood sampling performed every 30-min for 10-h for assessments of plasma peptide YY (PYY) and glucagon-like peptide-I (GLP-1). On day 7, the participants completed a free-living testing day at home in which additional servings of carbohydrate and fat-rich foods were provided, ad libitum, to assess 24-h food intake. Each 7-d dietary pattern period occurred during the follicular phase of the menstrual cycle; thus, there were 2- to 3-wk washout periods between dietary patterns. This study was registered at clinicaltrials.gov as NCT02614729.

### Dietary treatments

For 7 d/pattern, the participants were provided with eucaloric, isonitrogenous, and plant-based diets containing 3 daily meals (i.e., breakfast, lunch, and dinner), 28% of the daily energy/meal, and an evening snack containing 16% of daily energy (Table 1). The BEEF and PLANT patterns were matched for energy and macronutrient composition. Thus, the foundational part of both dietary patterns was plant-based and contained similar ingredients, recipes, and meal types. The diets varied only in the type of protein-rich foods provided. PLANT contained 100% of protein from plant sources, primarily from soy (BOCA Burger Ground Crumbles; Silken Tofu; and Smart Strips Steak Style, LightLife) and wheat gluten (Sweet Earth Seitan Strips). For BEEF, 60% of the plant-based protein foods were replaced with beef, as either flank steak or top round steak. All of the study foods were prepared, cooked, and packaged in the metabolic testing facility with each of the ingredients weighed to the nearest 0.1 g.

**TABLE 1:** Dietary characteristics of the study patterns

Daily intake	2 Servings of beef (BEEF)	0 Servings of beef (PLANT)
Energy content (kcal/d)	2010	1990
Total protein (g/d)	76.8	75.6
Beef protein (g/d)	45.0	0.0
(% of Total)	60	0
Plant proteins (g/d)	31.8	75.6
(% of Total)	40	100
Carbohydrates (g/d)	274.0	276.0
Dietary fiber (g/d)	26.2	31.7
Fat (g/d)	67.8	67.0
Beef, raw (oz/d)	7.5	0
(g/d)	207.0	0.0

The day before each 7-d testing period, participants picked up their meals and were provided with preparation instructions that outlined when meals were to be consumed. A food log was provided for participants to record compliance. Meal timing was specific to each participant’s habitual weekday breakfast mealtime followed by 4-, 8-, and 10-h mealtimes post breakfast, which corresponded to the consumption of lunch, dinner, and evening snacks, respectively. Supplemental Table 1 provides example meals across days 1–5 of the study. Meal types were similar across dietary patterns so that participants would be blinded to what pattern they were consuming each week. Participants were instructed to only consume foods provided to them during the intervention period, return all wrappers and uneaten foods to be weighed back, and document any deviations from this protocol such as not consuming all of the meal or eating foods not included in the study meal.

#### Day 6: 10-h clinical testing day

On day 6 of each pattern, the participants completed the following 10-h testing day. The participants arrived 1-h before breakfast, following a 10-h overnight fast, and were taken to a self-contained, comfortable, quiet, well-lit room to remain there throughout the testing day. The room contained a reclining chair, lamp, laptop (with Wi-Fi), and access to a bathroom. At −60 min, a catheter was inserted into the antecubital vein of the nondominant arm and kept patent via saline drip. At −30 min, computerized questionnaires assessing appetite and satiety were completed, and a fasting blood draw was performed. At time +0 min, the respective breakfast was consumed. Throughout the remainder of the day, the same computerized questionnaires were completed, and blood was drawn every 30 min. Lunch was consumed at +240 min, dinner was consumed at +480 min, and an evening snack was consumed at +600 min. Sensory testing was completed before and after each eating occasion to assess palatability and acceptability. At +660 min, the catheter was removed, and the participants left the facility.

##### Appetite and satiety questionnaires

Validated, computerized questionnaires assessing appetite (i.e., hunger, desire to eat, prospective food consumption) and satiety (i.e., fullness) were completed every 30 min throughout the 10-h testing day [22]. The questions were worded as “how strong is your feeling of” or “how strong is your desire” with anchors of “not at all” to “extremely.”

##### Repeated blood sampling and plasma analyses

Blood samples (4 mL/sample; 64 mL/testing day) were collected every 30 min throughout the 10-h testing day. The samples were collected in test tubes containing EDTA. Protease inhibitors (pefabloc SC and DPP-IV) were added to some of the tubes to reduce protein degradation. Within 10 min of collection, the samples were centrifuged at −4°C for 10 min. The plasma was separated and stored in microcentrifuge tubes at −80°C for future analysis. The plasma samples were assayed for plasma total PYY (Human PYY ELISA assay kits; EMD Millipore) and plasma total GLP-1 (Total GLP-1 ELISA assay kits; ALPCO).

#### Sensory testing

Computerized questionnaires assessing overall palatability (i.e., overall liking) and palatability subscales (i.e., appearance, aroma, flavor, texture) were completed after the first and last bite of each eating occasion throughout the 10-h testing day. The questions were worded as “how much do you like the” with anchors of “extremely dislike” to “extremely like.” An additional acceptability question was asked as “what is the likelihood that you would eat this on a daily basis if provided” with anchors of “low” to “high.”

#### ***Day 7: free-living testing day***

On day 7 of each pattern, each participant completed a free-living, ad libitum testing day. The participants were provided with and were required to consume the provided protein foods within each eating occasion based on the respective pattern. However, in addition to consuming the required protein, the participants were provided with additional servings of carbohydrate and fat-rich foods and were permitted to consume these, ad libitum, during the meal and snack eating occasions [23]. The additional foods at breakfast included banana bread, chocolate spread, and grapes. Additional lunch foods included potato salad and chocolate candy. Additional dinner foods were fried noodles and brownies. Additional snack foods included trail mix. All foods were weighed before and after the testing day for consumption of energy and macronutrient content.

### Data and statistical analyses

Summary statistics (mean ± SEM) were computed for all primary outcomes (daily fullness, 24-h food intake); secondary outcomes (daily hunger, desire to eat, prospective food consumption, PYY, and GLP-1); and exploratory outcomes (palatability and acceptability). Regarding the appetite and satiety responses, 10-h net incremental area under the curve (niAUC) was computed. Normality testing was performed on all study data. For all data normally distributed, paired *t*-tests were utilized to compare PLANT with BEEF on study outcomes. Because GLP-1 data were not normally distributed, Friedman's nonparametric test was performed. Analyses were conducted with the latest version of the Statistical Package for the Social Sciences (SPSS; 29.0; SPSS Inc.). *P* < 0.05 was considered statistically significant.

## Results

### Appetite and satiety

**FIGURE 1:**
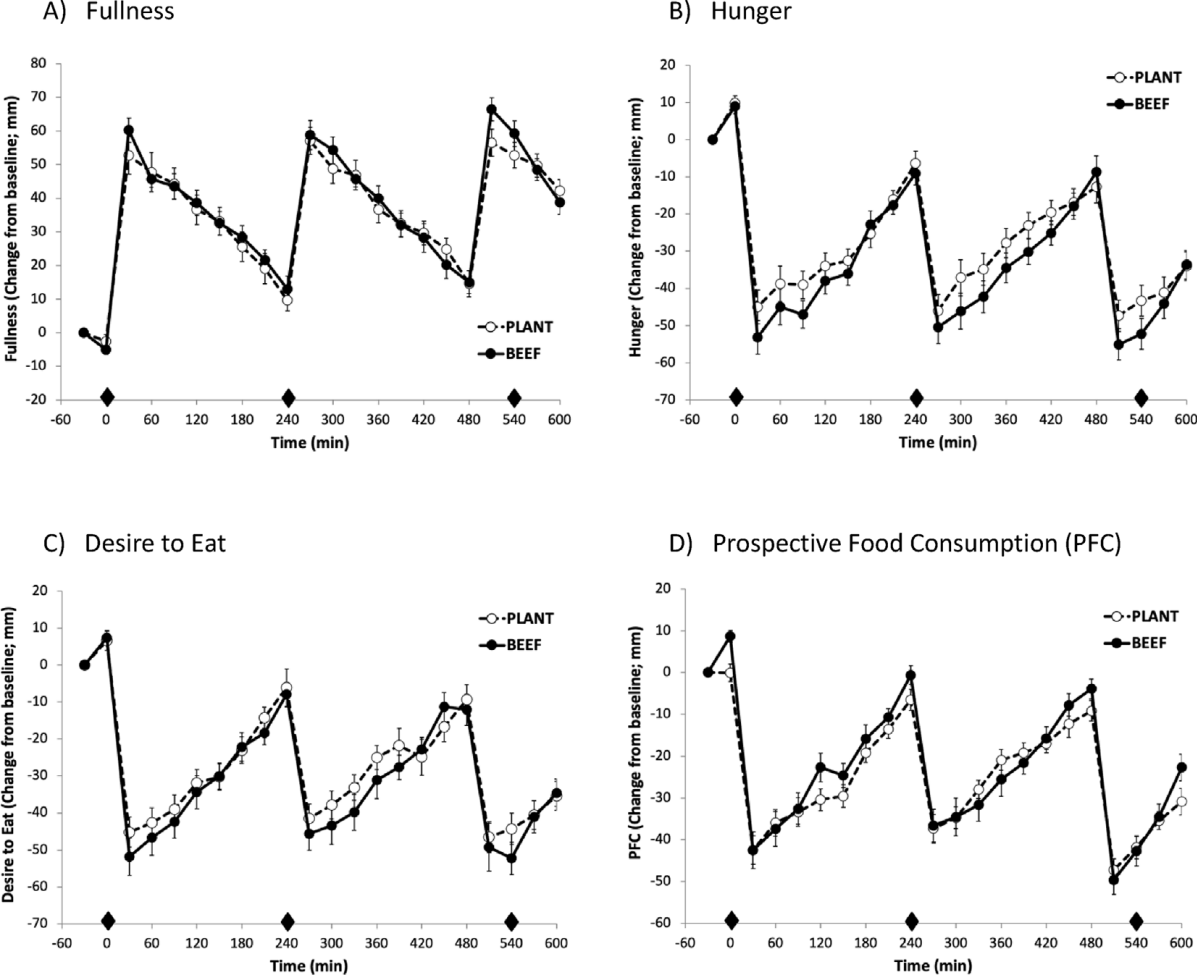
Postprandial perceived responses of appetite and satiety following the BEEF vs. PLANT dietary patterns in 17 healthy women with overweight. Diamond indicates when an eating occasion occurred (ie, breakfast, lunch, dinner).

**FIGURE 2:**
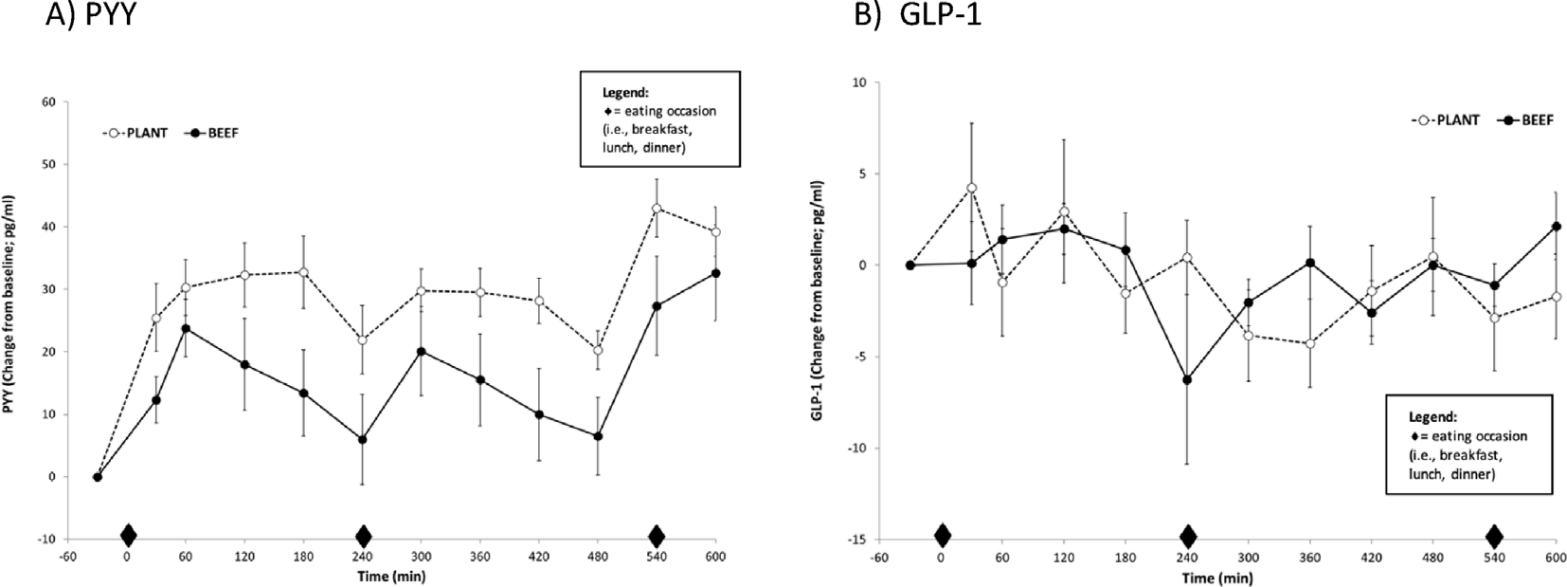
Postprandial hormonal responses following the BEEF vs. PLANT dietary patterns in 17 healthy women with overweight. Diamond indicates when an eating occasion occurred (ie, breakfast, lunch, dinner). GLP-1, glucagon-like peptide-1; PPY, peptide YY.

The perceived appetite and satiety responses across the 10-h testing day are shown in Figure 1A–D, and the satiety hormonal responses are shown in Figure 2A, B. Both dietary patterns led to pre- and postprandial fluctuations in perceived sensations and hormonal responses throughout the day. No difference in 10-h niAUC was detected between patterns for fullness (BEEF: 904 ± 472 compared with PLANT: 866 ± 333 mm × 600 min, *P* = 0.946); hunger (BEEF: 755 ± 357 compared with PLANT: 495 ± 321 mm × 600 min, *P* = 0.437); the desire to eat (BEEF: 344 ± 470 compared with PLANT: 539 ± 286 mm × 600 min, *P* = 0.688); and prospective food consumption (BEEF: 306 ± 281 compared with PLANT: 320 ± 176 mm × 600 min, *P* = 0.951). Similarly, 10-h PYY and GLP-1 niAUCs were not different between patterns (BEEF: 10,874 ± 5075 compared with PLANT: 18,954 ± 3082 pg/mL × 600 min, *P* = 0.088) and (BEEF: −9 ± 1056 compared with PLANT: −188 ± 1883 pg/mL × 600 min, *P* = 0.944), respectively.

### Ad libitum food intake

**FIGURE 3:**
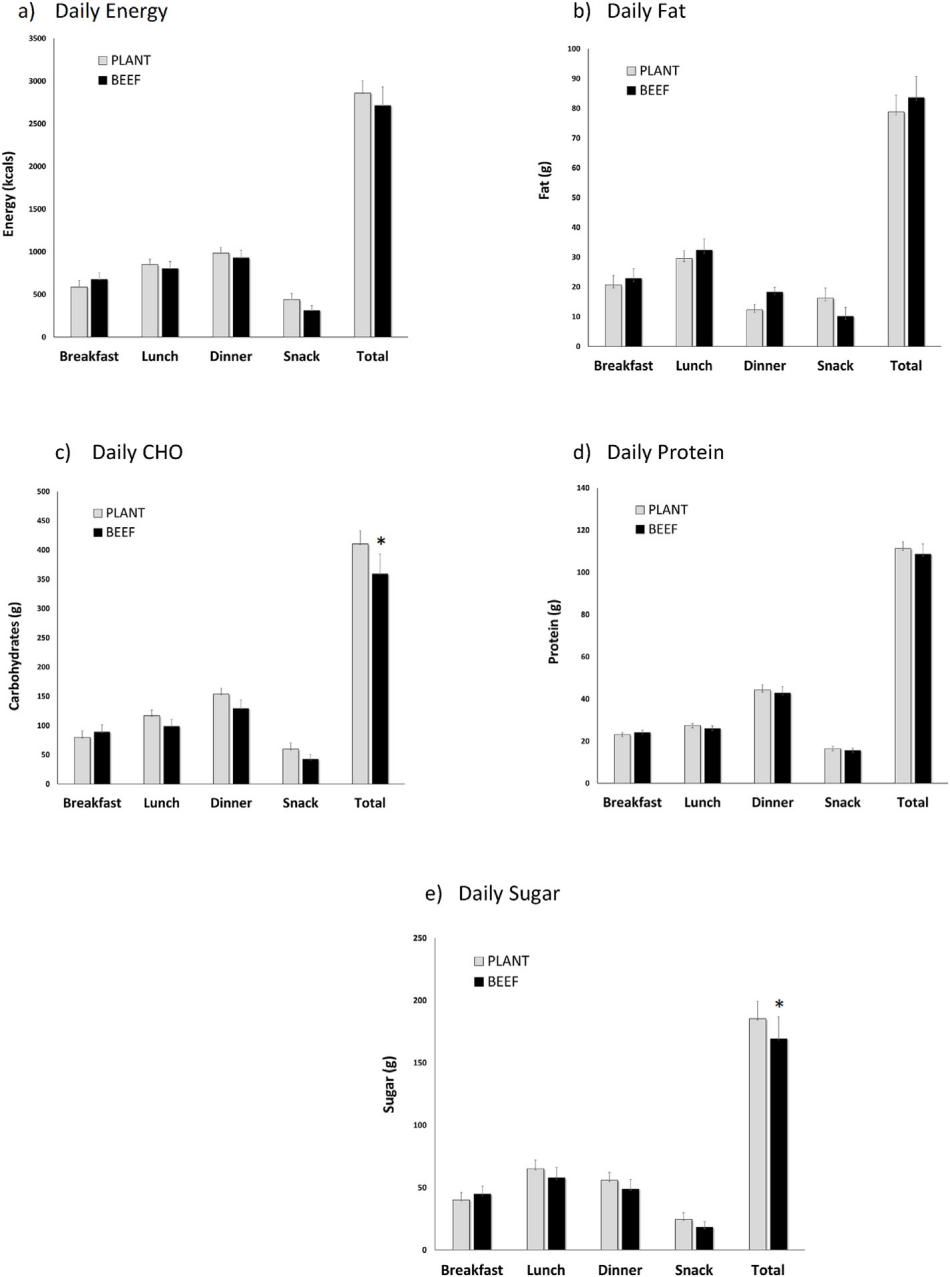
Twenty-four-hour food intake assessed during the free-living ad libitum testing day following the BEEF vs. PLANT dietary patterns in 17 healthy women with overweight. ∗Paired sample *t*-test, data reported as mean ± SEM; *P* < 0.05.

Twenty-four-hour energy and macronutrient content during the ad libitum testing day is shown in Figure 3. By design, total protein consumption did not differ between PLANT compared with BEEF. In terms of ad libitum consumption, 24-h energy and fat intake across the day did not differ between PLANT compared with BEEF. However, carbohydrate consumption was ∼12% (∼200 kcals) lower following BEEF compared with PLANT (*P* = 0.025), and sugar consumption was ∼9% (∼70 kcals) lower following BEEF compared with PLANT (*P* = 0.054).

### Sensory testing

The average palatability and acceptability ratings across all eating occasions are reported in Table 2. Compared with PLANT, BEEF was rated as having a higher flavor profile (*P* = 0.038), better texture (*P* = 0.040), and tended to elicit higher overall palatability (*P* = 0.071). In addition, the participants reported greater acceptability for BEEF compared with PLANT (*P* = 0.032).

**TABLE 2:** Sensory testing of the meals and snacks within the BEEF vs. PLANT patterns

Average responses across all eating occasions	2 Servings of beef (BEEF)	0 Servings of beef (PLANT)	Mean difference (BEEF–PLANT)	*P* value
Appearance (mm)	65.0 ± 2.8	60.0 ± 4.3	+5.0 ± 3.5	0.179
Aroma (mm)	62.2 ± 2.8	58.2 ± 3.9	+4.0 ± 3.1	0.217
Flavor (mm)	66.1 ± 3.5	58.0 ± 4.6	+8.2 ± 3.6	0.038
Texture (mm)	64.1 ± 3.0	55.1 ± 5.4	+9.0 ± 4.0	0.040
Overall Liking (mm)	68.4 ± 3.8	60.0 ± 5.5	+8.4 ± 4.3	0.071
Acceptability (mm)	65.4 ± 4.4	54.4 ± 6.0	+10.9 ± 4.6	0.032

Paired sample t-test, data reported as mean ± SEM.

## Discussion

We sought to examine whether short-term exposure to a diet containing fresh, lean beef would elicit greater satiety, reduce ad libitum food intake, and be more acceptable compared with a diet containing plant alternatives. Although no differences in satiety were detected between patterns, consuming a diet containing 2 servings of fresh, lean beef/day was more well liked and led to fewer sugar-rich carbohydrates consumed during a single ad libitum testing day compared with a diet containing plant protein alternatives. These findings highlight that meals containing lean beef are more well liked and promote healthier eating behavior during acute ad libitum testing compared with plant-based alternatives and challenge the current idea that animal and plant proteins are equivalent.

Although a large body of evidence exists comparing single animal- compared with plant-based protein isolates in beverage preload studies, only a few studies have compared animal compared with plant-based proteins in whole foods within a meal matrix [15], [16], [18], [20]. Of these, only 1 study [16] provided multiple animal- compared with plant-based meals over consecutive days – similar to the current study design.

Specifically, Neacsu et al. [16] provided higher-protein meals (∼50 g protein/meal) containing either animal-source foods, of chicken and beef, or plant-based alternatives, of soy protein and textured soy vegetable protein for 2 wk in men with overweight or obesity. Similar to the findings in the current study in women with overweight, no differences in appetitive or hormonal responses were detected between treatments; however, the animal protein-based meals were rated with higher pleasantness and acceptability compared with the plant-based protein versions. Because Neacsu et al. [16] did not include ad libitum feeding assessments, no comparisons can be made with respect to food choices.

In single test meal studies, all but 1 study [17], [19] report similar appetite, satiety, and subsequent food intake following plant- compared with animal-source protein meals [15], [17], [18], [20]. Those that include sensory testing reported greater palatability following the consumption of animal-source meals, primarily containing beef, compared with plant-based alternatives [18], [20]. The appetite, satiety, and palatability data from the current study align with the majority of published studies; notably, the lower ad libitum carbohydrate and sugar intakes following the BEEF compared with PLANT patterns are novel. Discrepant findings across studies may be due to differences in approach to assess ad libitum intake. In all previous studies, intake was assessed in a single, subsequent meal paradigm ∼3–4 h after the test meal [15], [16], [18], [19], [20]. In the current study, energy and macronutrient content were assessed within each test meal once the respective protein-rich food was consumed. Thus, our findings suggest that consuming protein-rich foods, varying in protein quality, impacts the amount and type of carbohydrate and sugar-rich foods consumed. Given the short duration of the dietary interventions within this study, we were unable to assess any long-term changes in eating behavior, food choice, and diet quality. However, if this persistent behavior were to continue, there could be health consequences not typically considered when recommending these types of protein exchanges as healthier alternatives.

### Considerations

Although power analyses were originally performed to assess sample size estimates using daily fullness and 24-h food intake data from previous studies [24], [25], the dietary comparisons were between normal compared with higher protein versions, not plant- compared with animal-source foods. Thus, it is possible that the current study was underpowered to detect protein source differences within the primary outcomes. However, the sample size included in this study (*n* = 17) is comparable with most crossover studies assessing protein source differences [15], [16], [18], [19], [20].

As is with all acute, controlled-feeding trials, whether findings from this study would translate to changes in a free-living population over the long-term is unclear. However, the study was longer than most controlled-feeding studies of this nature, including a 7-d treatment design that provided all meals and snacks across each day.

We sought to examine dietary patterns that resemble the typical American diet. As such, we included protein intakes of ∼15% of daily intake as protein (∼20 g protein/meal) in the BEEF and PLANT patterns. Data from our laboratory and others support the consumption of ∼30 g high-quality, animal-source protein/eating occasion to elicit an effect on satiety when compared with lower protein quantities [26]. Thus, the possibility that the lack of satiety differences between treatments may have been a result of subthreshold quantities of total protein provided exists.

Most studies assessing appetite, satiety, and eating behavior complete prestudy sensory testing to match for palatability and acceptability of the test foods and meals provided to the study participants. We chose not to match for palatability and acceptability and instead completed sensory testing during the study visits. With this design, we were able to evaluate whether the DGA recommendations to replace animal-source foods, such as fresh, lean beef, with plant alternatives is well liked and feasible for adults in the United States. However, allowing palatability to vary between dietary patterns may have influenced the amount and type of foods consumed during the ad libitum testing day because palatability is known to impact food choice, food preference, and the amount of food consumed within an eating occasion [27]. Additional work is needed to identify which behavioral and/or sensory factors elicit the greatest effects on appetite, satiety, and subsequent food intake.

In conclusion, the short-term consumption of animal-based protein-rich foods, such as fresh, lean beef, did not impact satiety but was more well liked, acceptable, and promoted healthier eating behaviors during a single-day exposure compared with plant-based alternatives in women with overweight. However, additional research is needed to identify the long-term effects of replacing animal-sourced protein foods with plant-based alternatives on diet quality and daily food intake.

## Author contributions

The authors’ responsibilities were as follows – HJL, JAG: designed the research project and completed all study procedures; MLB: completed all hormonal analyses; HJL, MLB: completed all statistical analyses; MLB: developed the ﬁrst draft of the manuscript; HJL, JAG, MLB: substantially contributed to the completion of the manuscript; and all authors: read and approved the ﬁnal manuscript.

## Conflict of interest

MLB and JAG have no conflicts of interest to report. HJL is on the speaker bureau of the National Cattlemen’s Beef Association and has received funding from the Beef Checkoff. The opinions or assertions contained herein are the private views of the authors and are not to be construed as official or as reflecting the views of the Army or the Department of Defense. Any citations of commercial organizations and trade names in this report do not constitute an official Department of the Army endorsement of approval of the products or services of these organizations.

## Funding

The Beef Checkoff supplied research funding for recruitment and screening, study procedures, hormones analyses, graduate student support, and participant stipends.
