# Supplementary material for: A Diet Containing Animal Source Protein as Fresh, Lean Beef Is More Well Liked and Promotes Healthier Eating Behavior Compared with Plant-Based Alternatives in Women with Overweight
Source: Curr Dev Nutr. 2024 Jul 14;8(8):104415. doi: 10.1016/j.cdnut.2024.104415 (PMC11367550; doi:10.1016/j.cdnut.2024.104415)
Supplement: multimedia component [file mmc1.docx]

Supplemental Table 1: Example meals across days 1-5 for each dietary pattern

| **Day 1**  **Foods** | **Day 2**  **Foods** | **Day 3**  **Foods** | **Day 4**  **Foods** | **Day 5**  **Foods** |
| --- | --- | --- | --- | --- |
| *Breakfast:*  Eggs & Hash  Fig Newtons  *Lunch:*  Steak Sandwich  Chips  Grapes  *Dinner:*  Pot Pie  Caramel Candy  *Snack:*  Pizza Wedge  Peppermints | *Breakfast:*  Quesadilla  Pineapples  *Lunch:*  Stir-fry  Mints  *Dinner:*  Roast Meat & Gravy  Mashed Potatoes  Apple Pie  Mints  *Snack:*  Hummus  Jerky  Vegetables  Pretzels  Peppermints | *Breakfast:*  Fajita  Dried Fruit  *Lunch:*  Meatloaf  Vegetables  Apple Crisp  *Dinner:*  Enchilada  Chocolate Candy  *Snack:*  Meat Pocket  Yogurt- covered Raisins | *Breakfast:*  Breakfast Pizza  Peaches  *Lunch:*  Stir-fry  Chocolate Cereal  *Dinner:*  Lasagna  Brownie Cookies  *Snack:*  Hummus  Jerky  Vegetables  Pretzels  Peppermints | *Breakfast:*  Eggs & Hash  Mandarin Oranges  *Lunch:*  Steak Salad  Sweet Potato Fries  Peppermints  *Dinner:*  Pot Pie  Applesauce  Caramel Candy  *Snack:*  Pizza Wedge  Peppermints |
